# Editorial: Medical Education in Psychiatry

**DOI:** 10.3389/fpsyt.2021.764567

**Published:** 2021-10-04

**Authors:** Doron Amsalem, Robbert Duvivier, Andrés Martin

**Affiliations:** ^1^New York State Psychiatric Institute and Department of Psychiatry, Columbia University Vagelos College of Physicians & Surgeons, New York, NY, United States; ^2^Center for Educational Development and Research in Health Sciences (CEDAR), LEARN, University Medical Center Groningen, Groningen, Netherlands; ^3^Parnassia Psychiatric Institute, The Hague, Netherlands; ^4^Child Study Center, School of Medicine, Yale University, New Haven, CT, United States

**Keywords:** medical education, online platform, psychiatry, stigma, simulation, simulated patients, training

Medical education in psychiatry stands to gain from educational innovations but has been slow on the uptake. For example, although simulated-patient (SPs)-based methodology has a rich history in medical education, its use in psychiatry has only emerged more recently. Simulation is particularly effective in bridging the gap between clinical practice and education. The use of professional actors as SPs offers advantages that have been well-reviewed in the literature (1). Practicing pre-specified clinical scenarios in a safe environment carries no risk of harm by inexperienced students or residents. It provides the trainer and trainee the opportunity to focus their attention on relevant objectives for specific training needs (2). In addition, the unprecedented nature and scale of the COVID-19 outbreak created a rare opportunity to shift traditional in-person SP training to online platforms. This approach provides several important advantages: greater ease of dissemination to large audiences, training consistency across different geographic locations; interaction in real time with content experts and fellow learners; and logistic ease in securing curricular content and experienced faculty (3).

Stigma toward psychiatry and people with mental illness is prevalent. Stigma among physicians contributes to poor medical treatment and outcomes, including earlier death in patients with psychiatric disorders like schizophrenia (4, 5). Starting in medical school, and despite overwhelming evidence to the contrary, students commonly report that psychiatric treatments have little effect or utility and view the specialty as a non-evidence-based, unscientific, barely medical discipline (6). Interventions to reduce stigma, including online programs that embed SPs and principles of direct social contact (7), have the potential to change this biased outlook early in medical education.

There is an urgent need to bring state-of-the-art teaching and educational approaches into all domains of psychiatric education: from the undergraduate level in medical school, through graduate training in residency and fellowships, and into the lifelong learning of community and academic practice. The number of peer-reviewed scholarly publications dedicated to medical education in psychiatry is relatively limited, particularly compared to disciplines such as internal medicine and surgery. Thus, rigorous publications in peer-reviewed outlets are a modifiable factor that could enhance interest in, and foster the development of this scholarly domain. To that end, we invited contributions of work at the interface of medical education and psychiatry for a special issue of *Frontiers in Psychiatry*.

Several studies in this issue investigate the intersection between the use of SPs and delivery via synchronized videoconferencing. In an example of innovative use of SPs, Martin et al. describe the first implementation, in child psychiatry, of Co-Constructive Patient Simulation (CCPS), an experiential approach that fosters autonomous, meaningful, and individually tailored learning opportunities generalizable to any medical specialty. Drozdowicz et al. developed a didactic using SPs that addresses sexual health specifically pertinent to psychiatrists working with adolescents and their families. They found that video-based examples using SPs measurably improved factual knowledge—and decreased discomfort in addressing the topic in routine clinical practice.

Three studies in the collection use multi-component approaches, each involving SPs, to address very different learning objectives. In another example in child psychiatry education, Curtin et al. demonstrated the efficacy of an interactive online curriculum designed to equip learners with practical tools to care for patients with pediatric depression and suicidality. In turn, Rodríguez-Rivas et al. found a positive impact in reducing mental health stigma after participation in an online, multi-pronged program that incorporates project-based learning, clinical simulations with SPs, and e-contact with individuals with schizophrenia. In the third of these studies, a non-inferiority trial incorporating interviews with SPs, Rauch et al. found no difference in grades in an objective structured clinical examination (OSCE) between students learning on-site vs. online.

Altough there is a rich scholarship on best practices to deliever challenging news, there are very few applications specific to psychiatry. Two of the articles in this series contribute to this gap in the literature. In a mixed methods study, Brand-Gothelf et al. describe stressful factors associated with sharing a diagnosis of schizophrenia (vs. ADHD) in the practice of child and adolescent psychiatry. Their findings contribute to a better understanding of the critically important yet inadequately addressed topic of effective and compassionate diagnosis delivery in psychiatry. To help address that training gap, Amsalem et al. developed, implemented, and evaluated the effectiveness of an SP-based training module to improve psychiatric residents' clinical communication skills in delivering difficult news. The training module proved helpful in improving objectively measured communication skills and enhanced participants' subjective sense of confidence in their clinical skills.

Attesting to the growth, broad dissemination, and creative applications of simulation-based training in psychiatric education, Piot et al. present the most comprehensive review on the topic to date. The holistic and reflective nature of simulation aligns with the rich humanistic tradition of psychiatry and offers unique opportunities to support learners' clinical skills and develop and refine workforce competencies.

Three articles in the series focus on program developmend in psychiatric education. First, Calhoun et al. conducted a qualitative study to explore the impact and active components of a novel educational initiative designed to prepare physician-scientists for independent careers in investigating and treating childhood psychiatric disorders. They developed a heuristic model of practical relevance to other training initiatives within psychiatry—and beyond. Second, Kulkarni et al. developed a process to identify and prioritize key attributes to inform trainee selection into a psychiatry residency program. Their approach has additional programmatic implications, including curriculum, faculty, and mission and vision development. Finally, Wang et al. examined the role of perceived stress on depressive symptoms among medical students. Their findings emphasize the need to identify students with high achievement goal-orientation in learning environments that explicitly or unwittinlgy foster fierce competion. Providing such learners with the appropriate supportive services can help them manage stress and mitigate or prevent clinical depression or dropping out of medical school.

The 11 articles in this special collection encompasss contributions from over a dozen countries spread acrosss four continents. Their topic spectrum is broad and has practical applicability for educators in mental health working at undergraduate, graduate, or lifelong/continuous medical education levels. We hope that this thematic series will contribute to necessary and welcome exchanges between psychiatric training and best practices in medical education. Progress at this exciting interface could accelerate the growth of visibile and impactful publications dedicated to advancing medical education in psychiatry. The ultimate goal of these synergistic efforts is to improve the lives of so many who are impacted by mental illnesses.

## Author Contributions

DA, RD, and AM contributed to the final version of the manuscript. All authors contributed to the article and approved the submitted version.

## Conflict of Interest

The authors declare that the research was conducted in the absence of any commercial or financial relationships that could be construed as a potential conflict of interest.

## Publisher's Note

All claims expressed in this article are solely those of the authors and do not necessarily represent those of their affiliated organizations, or those of the publisher, the editors and the reviewers. Any product that may be evaluated in this article, or claim that may be made by its manufacturer, is not guaranteed or endorsed by the publisher.
